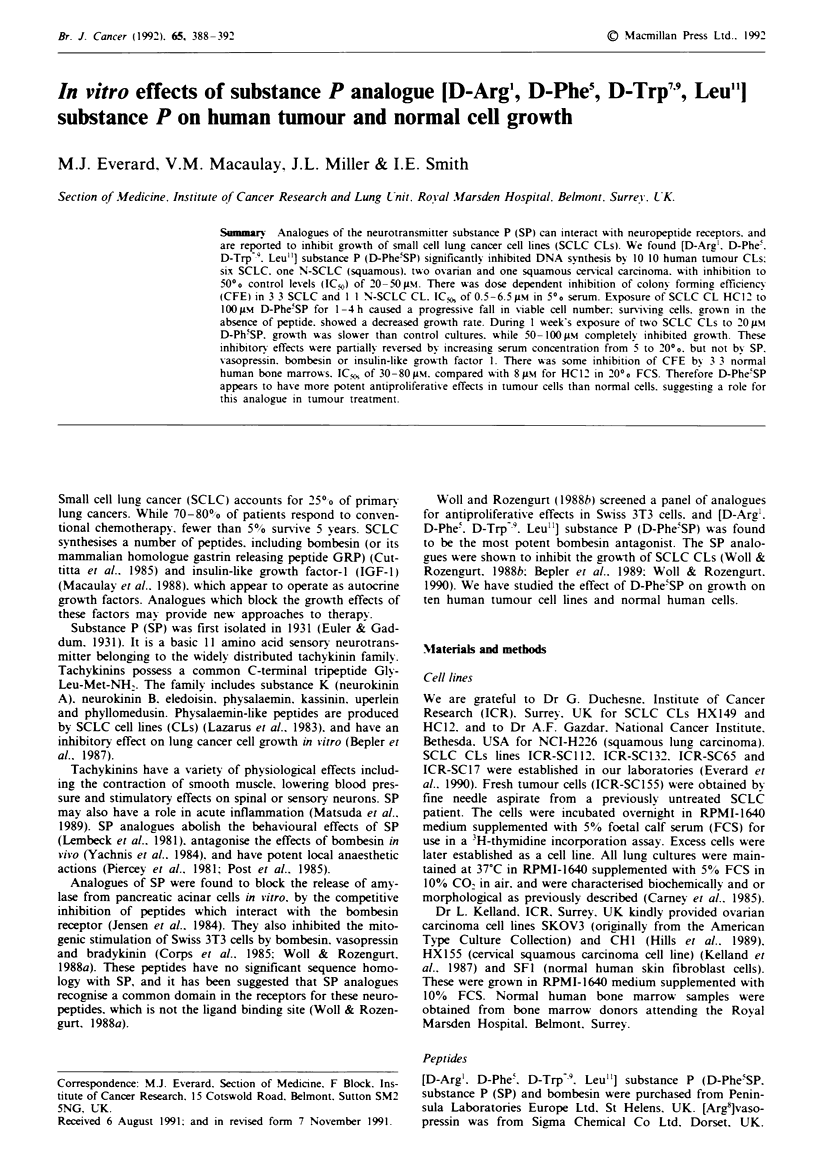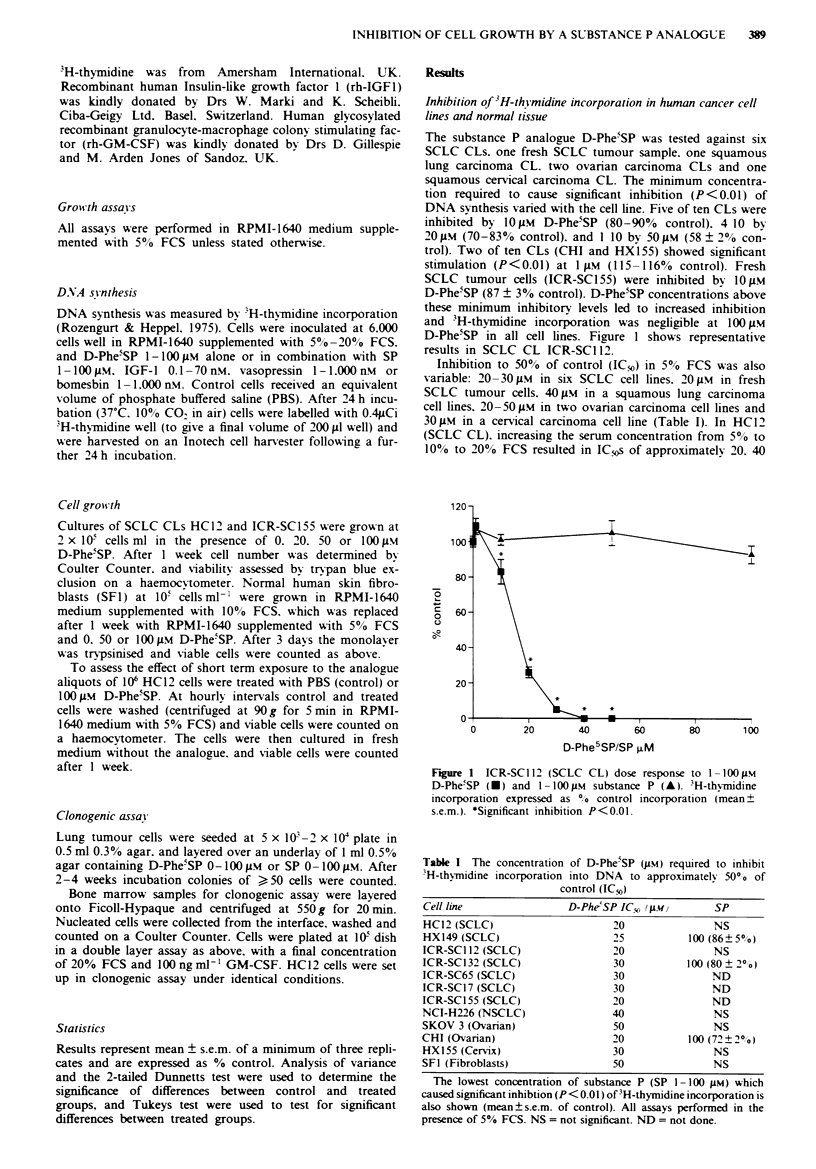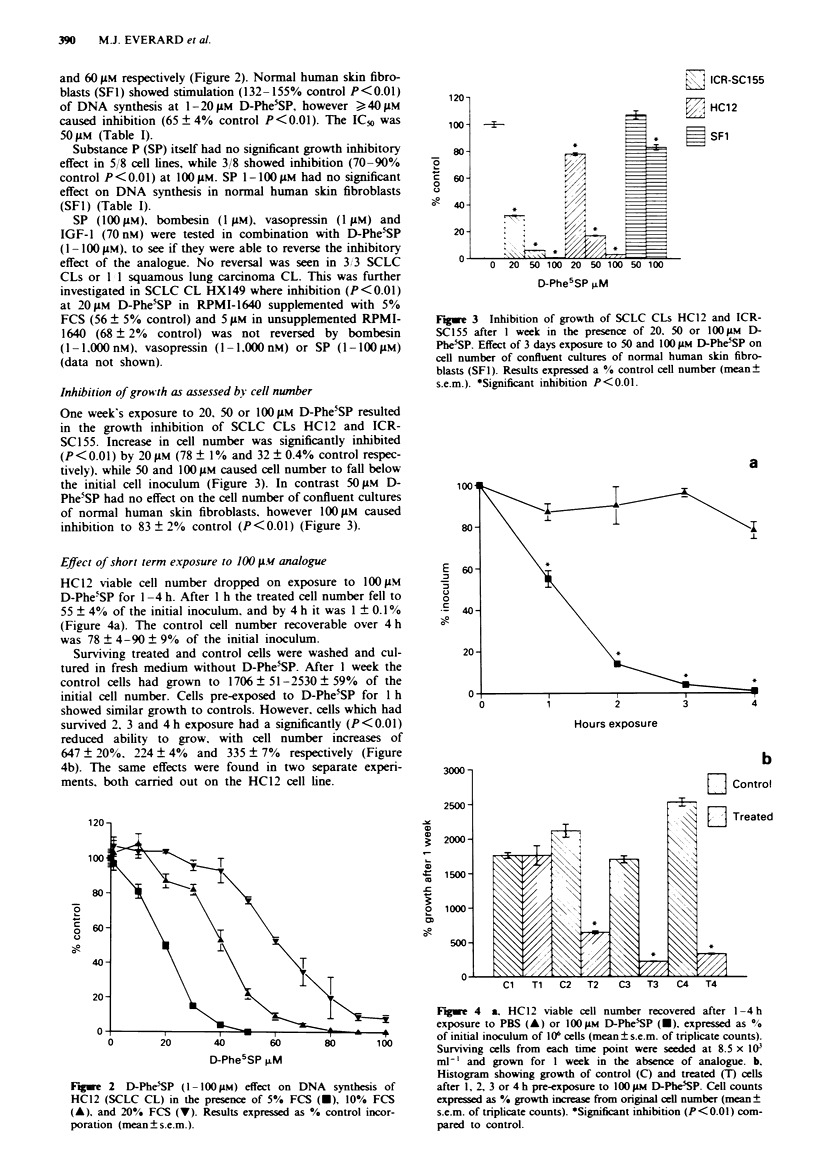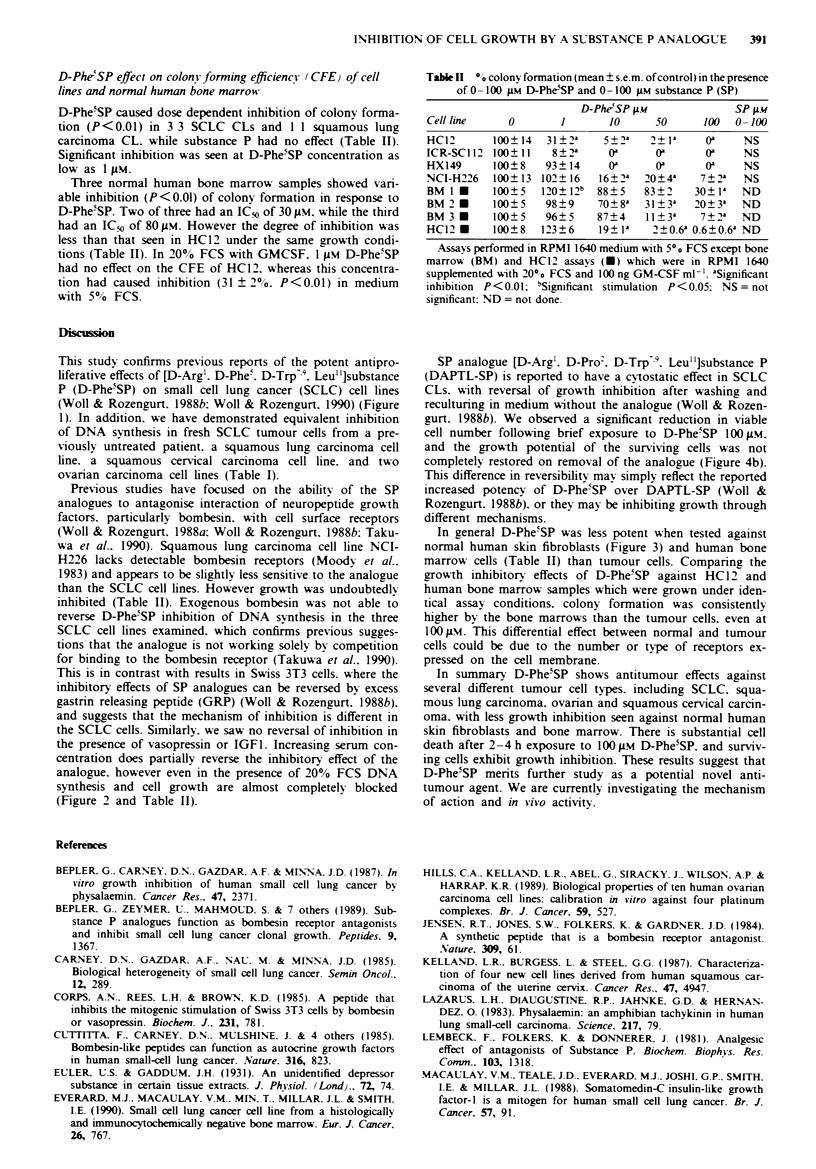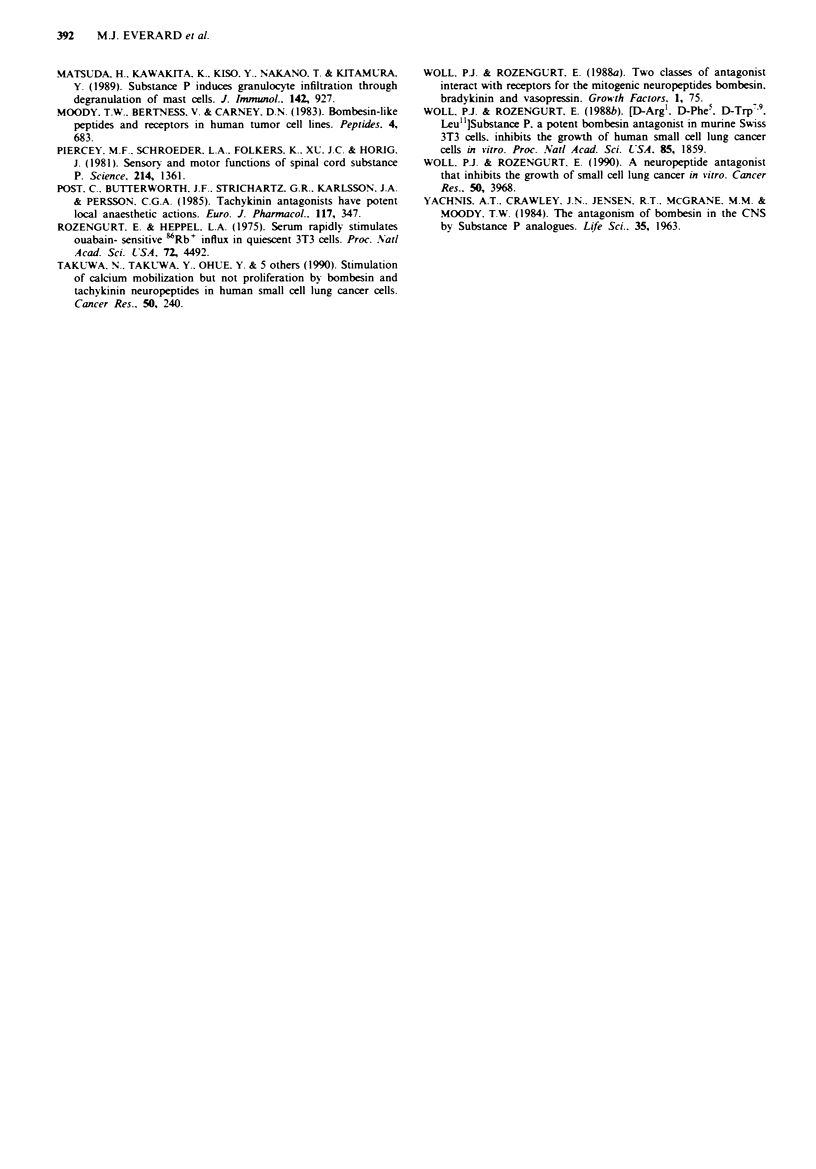# In vitro effects of substance P analogue [D-Arg1, D-Phe5, D-Trp7,9, Leu11] substance P on human tumour and normal cell growth.

**DOI:** 10.1038/bjc.1992.78

**Published:** 1992-03

**Authors:** M. J. Everard, V. M. Macaulay, J. L. Miller, I. E. Smith

**Affiliations:** Section of Medicine, Royal Marsden Hospital, Belmont, Surrey, UK.

## Abstract

Analogues of the neurotransmitter substance P (SP) can interact with neuropeptide receptors, and are reported to inhibit growth of small cell lung cancer cell lines (SCLC CLs). We found [D-Arg1, D-Phe5, D-Trp7,9, Leu11] substance P (D-Phe5SP) significantly inhibited DNA synthesis by 10/10 human tumour CLs; six SCLC, one N-SCLC (squamous), two ovarian and one squamous cervical carcinoma, with inhibition to 50% control levels (IC50) of 20-50 microM. There was dose dependent inhibition of colony forming efficiency (CFE) in 3/3 SCLC and 1/1 N-SCLC CL, IC50s of 0.5-6.5 microM in 5% serum. Exposure of SCLC CL HC12 to 100 microM D-Phe5SP for 1-4 h caused a progressive fall in viable cell number; surviving cells, grown in the absence of peptide, showed a decreased growth rate. During 1 week's exposure of two SCLC CLs to 20 microM D-Ph5SP, growth was slower than control cultures, while 50-100 microM completely inhibited growth. These inhibitory effects were partially reversed by increasing serum concentration from 5 to 20%, but not by SP, vasopressin, bombesin or insulin-like growth factor 1. There was some inhibition of CFE by 3/3 normal human bone marrows, IC50s of 30-80 microM, compared with 8 microM for HC12 in 20% FCS. Therefore D-Phe5SP appears to have more potent antiproliferative effects in tumour cells than normal cells, suggesting a role for this analogue in tumour treatment.


*Br. J. Cancer* (1992), **65**, 388–392 © Macmillan Press Ltd., 1992

### *In vitro* effects of substance *P* analogue [D-Arg$^{1}$, D-Phe$^{5}$, D-Trp$^{7,9}$, Leu$^{11}$] substance *P* on human tumour and normal cell growth

M.J. Everard, V.M. Macaulay, J.L. Miller & I.E. Smith

*Section of Medicine, Institute of Cancer Research and Lung Unit, Royal Marsden Hospital, Belmont, Surrey, UK.*

**Summary** Analogues of the neurotransmitter substance P (SP) can interact with neuropeptide receptors, and are reported to inhibit growth of small cell lung cancer cell lines (SCLC CLs). We found [D-Arg$^{1}$, D-Phe$^{5}$, D-Trp$^{7,9}$, Leu$^{11}$] substance P (D-Phe$^{5}$SP) significantly inhibited DNA synthesis by 10/10 human tumour CLs: six SCLC, one N-SCLC (squamous), two ovarian and one squamous cervical carcinoma, with inhibition to 50% control levels ($IC_{50}$) of 20–50 µM. There was dose dependent inhibition of colony forming efficiency (CFE) in 3/3 SCLC and 1/1 N-SCLC CL, $IC_{50}$s of 0.5–6.5 µM in 5% serum. Exposure of SCLC CL HC12 to 100 µM D-Phe$^{5}$SP for 1–4 h caused a progressive fall in viable cell number; surviving cells, grown in the absence of peptide, showed a decreased growth rate. During 1 week's exposure of two SCLC CLs to 20 µM D-Ph$^{5}$SP, growth was slower than control cultures, while 50–100 µM completely inhibited growth. These inhibitory effects were partially reversed by increasing serum concentration from 5 to 20%, but not by SP, vasopressin, bombesin or insulin-like growth factor 1. There was some inhibition of CFE by 3/3 normal human bone marrows, $IC_{50}$s of 30–80 µM, compared with 8 µM for HC12 in 20% FCS. Therefore D-Phe$^{5}$SP appears to have more potent antiproliferative effects in tumour cells than normal cells, suggesting a role for this analogue in tumour treatment.

Small cell lung cancer (SCLC) accounts for 25% of primary lung cancers. While 70–80% of patients respond to conventional chemotherapy, fewer than 5% survive 5 years. SCLC synthesises a number of peptides, including bombesin (or its mammalian homologue gastrin releasing peptide GRP) (Cuttitta *et al.*, 1985) and insulin-like growth factor-1 (IGF-1) (Macaulay *et al.*, 1988), which appear to operate as autocrine growth factors. Analogues which block the growth effects of these factors may provide new approaches to therapy.

Substance P (SP) was first isolated in 1931 (Euler & Gaddum, 1931). It is a basic 11 amino acid sensory neurotransmitter belonging to the widely distributed tachykinin family. Tachykinins possess a common C-terminal tripeptide Gly-Leu-Met-$NH_2$. The family includes substance K (neurokinin A), neurokinin B, eledoisin, physalaemin, kassinin, uperlein and phyllomedusin. Physalaemin-like peptides are produced by SCLC cell lines (CLs) (Lazarus *et al.*, 1983), and have an inhibitory effect on lung cancer cell growth *in vitro* (Bepler *et al.*, 1987).

Tachykinins have a variety of physiological effects including the contraction of smooth muscle, lowering blood pressure and stimulatory effects on spinal or sensory neurons. SP may also have a role in acute inflammation (Matsuda *et al.*, 1989). SP analogues abolish the behavioural effects of SP (Lembeck *et al.*, 1981), antagonise the effects of bombesin *in vivo* (Yachnis *et al.*, 1984), and have potent local anaesthetic actions (Piercey *et al.*, 1981; Post *et al.*, 1985).

Analogues of SP were found to block the release of amylase from pancreatic acinar cells *in vitro*, by the competitive inhibition of peptides which interact with the bombesin receptor (Jensen *et al.*, 1984). They also inhibited the mitogenic stimulation of Swiss 3T3 cells by bombesin, vasopressin and bradykinin (Corps *et al.*, 1985; Woll & Rozengurt, 1988*a*). These peptides have no significant sequence homology with SP, and it has been suggested that SP analogues recognise a common domain in the receptors for these neuropeptides, which is not the ligand binding site (Woll & Rozengurt, 1988*a*).

Woll and Rozengurt (1988*b*) screened a panel of analogues for antiproliferative effects in Swiss 3T3 cells, and [D-Arg$^{1}$, D-Phe$^{5}$, D-Trp$^{7,9}$, Leu$^{11}$] substance P (D-Phe$^{5}$SP) was found to be the most potent bombesin antagonist. The SP analogues were shown to inhibit the growth of SCLC CLs (Woll & Rozengurt, 1988*b*; Bepler *et al.*, 1989; Woll & Rozengurt, 1990). We have studied the effect of D-Phe$^{5}$SP on growth on ten human tumour cell lines and normal human cells.

### Materials and methods

### *Cell lines*

We are grateful to Dr G. Duchesne, Institute of Cancer Research (ICR), Surrey, UK for SCLC CLs HX149 and HC12, and to Dr A.F. Gazdar, National Cancer Institute, Bethesda, USA for NCI-H226 (squamous lung carcinoma). SCLC CLs lines ICR-SC112, ICR-SC132, ICR-SC65 and ICR-SC17 were established in our laboratories (Everard *et al.*, 1990). Fresh tumour cells (ICR-SC155) were obtained by fine needle aspirate from a previously untreated SCLC patient. The cells were incubated overnight in RPMI-1640 medium supplemented with 5% foetal calf serum (FCS) for use in a $^{3}$H-thymidine incorporation assay. Excess cells were later established as a cell line. All lung cultures were maintained at 37°C in RPMI-1640 supplemented with 5% FCS in 10% $CO_2$ in air, and were characterised biochemically and or morphological as previously described (Carney *et al.*, 1985).

Dr L. Kelland, ICR, Surrey, UK kindly provided ovarian carcinoma cell lines SKOV3 (originally from the American Type Culture Collection) and CH1 (Hills *et al.*, 1989), HX155 (cervical squamous carcinoma cell line) (Kelland *et al.*, 1987) and SF1 (normal human skin fibroblast cells). These were grown in RPMI-1640 medium supplemented with 10% FCS. Normal human bone marrow samples were obtained from bone marrow donors attending the Royal Marsden Hospital, Belmont, Surrey.

### *Peptides*

[D-Arg$^{1}$, D-Phe$^{5}$, D-Trp$^{7,9}$, Leu$^{11}$] substance P (D-Phe$^{5}$SP, substance P (SP) and bombesin were purchased from Peninsula Laboratories Europe Ltd, St Helens, UK. [Arg$^{8}$]vasopressin was from Sigma Chemical Co Ltd, Dorset, UK.

Correspondence: M.J. Everard, Section of Medicine, F Block, Institute of Cancer Research, 15 Cotswold Road, Belmont, Sutton SM2 5NG, UK.
Received 6 August 1991; and in revised form 7 November 1991.

$^3$H-thymidine was from Amersham International, UK. Recombinant human Insulin-like growth factor 1 (rh-IGF1) was kindly donated by Drs W. Marki and K. Scheibli, Ciba-Geigy Ltd, Basel, Switzerland. Human glycosylated recombinant granulocyte-macrophage colony stimulating factor (rh-GM-CSF) was kindly donated by Drs D. Gillespie and M. Arden Jones of Sandoz, UK.

*Growth assays*

All assays were performed in RPMI-1640 medium supplemented with 5% FCS unless stated otherwise.

*DNA synthesis*

DNA synthesis was measured by $^3$H-thymidine incorporation (Rozengurt & Heppel, 1975). Cells were inoculated at 6,000 cells well in RPMI-1640 supplemented with 5%-20% FCS, and D-Phe$^5$SP 1-100 μM alone or in combination with SP 1-100 μM, IGF-1 0.1-70 nM, vasopressin 1-1,000 nM or bomesbin 1-1,000 nM. Control cells received an equivalent volume of phosphate buffered saline (PBS). After 24 h incubation (37°C, 10% $CO_2$ in air) cells were labelled with 0.4μCi $^3$H-thymidine well (to give a final volume of 200 μl well) and were harvested on an Inotech cell harvester following a further 24 h incubation.

*Cell growth*

Cultures of SCLC CLs HC12 and ICR-SC155 were grown at $2 \times 10^5$ cells ml in the presence of 0, 20, 50 or 100 μM D-Phe$^5$SP. After 1 week cell number was determined by Coulter Counter, and viability assessed by trypan blue exclusion on a haemocytometer. Normal human skin fibroblasts (SF1) at $10^5$ cells $ml^{-1}$ were grown in RPMI-1640 medium supplemented with 10% FCS, which was replaced after 1 week with RPMI-1640 supplemented with 5% FCS and 0, 50 or 100 μM D-Phe$^5$SP. After 3 days the monolayer was trypsinised and viable cells were counted as above.

To assess the effect of short term exposure to the analogue aliquots of $10^6$ HC12 cells were treated with PBS (control) or 100 μM D-Phe$^5$SP. At hourly intervals control and treated cells were washed (centrifuged at 90 *g* for 5 min in RPMI-1640 medium with 5% FCS) and viable cells were counted on a haemocytometer. The cells were then cultured in fresh medium without the analogue, and viable cells were counted after 1 week.

*Clonogenic assay*

Lung tumour cells were seeded at $5 \times 10^3 - 2 \times 10^4$ plate in 0.5 ml 0.3% agar, and layered over an underlay of 1 ml 0.5% agar containing D-Phe$^5$SP 0-100 μM or SP 0-100 μM. After 2-4 weeks incubation colonies of ⩾50 cells were counted.

Bone marrow samples for clonogenic assay were layered onto Ficoll-Hypaque and centrifuged at 550 *g* for 20 min. Nucleated cells were collected from the interface, washed and counted on a Coulter Counter. Cells were plated at $10^5$ dish in a double layer assay as above, with a final concentration of 20% FCS and 100 ng $ml^{-1}$ GM-CSF. HC12 cells were set up in clonogenic assay under identical conditions.

*Statistics*

Results represent mean ± s.e.m. of a minimum of three replicates and are expressed as % control. Analysis of variance and the 2-tailed Dunnetts test were used to determine the significance of differences between control and treated groups, and Tukeys test were used to test for significant differences between treated groups.

### Results

*Inhibition of $^3$H-thymidine incorporation in human cancer cell lines and normal tissue*

The substance P analogue D-Phe$^5$SP was tested against six SCLC CLs, one fresh SCLC tumour sample, one squamous lung carcinoma CL, two ovarian carcinoma CLs and one squamous cervical carcinoma CL. The minimum concentration required to cause significant inhibition ($P < 0.01$) of DNA synthesis varied with the cell line. Five of ten CLs were inhibited by 10 μM D-Phe$^5$SP (80-90% control), 4 10 by 20 μM (70-83% control), and 1 10 by 50 μM (58 ± 2% control). Two of ten CLs (CHI and HX155) showed significant stimulation ($P < 0.01$) at 1 μM (115-116% control). Fresh SCLC tumour cells (ICR-SC155) were inhibited by 10 μM D-Phe$^5$SP (87 ± 3% control). D-Phe$^5$SP concentrations above these minimum inhibitory levels led to increased inhibition and $^3$H-thymidine incorporation was negligible at 100 μM D-Phe$^5$SP in all cell lines. Figure 1 shows representative results in SCLC CL ICR-SC112.

Inhibition to 50% of control ($IC_{50}$) in 5% FCS was also variable: 20-30 μM in six SCLC cell lines, 20 μM in fresh SCLC tumour cells, 40 μM in a squamous lung carcinoma cell lines, 20-50 μM in two ovarian carcinoma cell lines and 30 μM in a cervical carcinoma cell line (Table I). In HC12 (SCLC CL), increasing the serum concentration from 5% to 10% to 20% FCS resulted in $IC_{50}$s of approximately 20, 40

**Figure 1** ICR-SC112 (SCLC CL) dose response to 1-100 μM D-Phe$^5$SP (■) and 1-100 μM substance P (▲). $^3$H-thymidine incorporation expressed as % control incorporation (mean ± s.e.m.). *Significant inhibition $P < 0.01$.

**Table I** The concentration of D-Phe$^5$SP (μM) required to inhibit $^3$H-thymidine incorporation into DNA to approximately 50% of control ($IC_{50}$)

| *Cell line* | *D-Phe$^5$SP $IC_{50}$ (μM)* | *SP* |
|---|---|---|
| HC12 (SCLC) | 20 | NS |
| HX149 (SCLC) | 25 | 100 (86 ± 5%) |
| ICR-SC112 (SCLC) | 20 | NS |
| ICR-SC132 (SCLC) | 30 | 100 (80 ± 2%) |
| ICR-SC65 (SCLC) | 30 | ND |
| ICR-SC17 (SCLC) | 30 | ND |
| ICR-SC155 (SCLC) | 20 | ND |
| NCI-H226 (NSCLC) | 40 | NS |
| SKOV 3 (Ovarian) | 50 | NS |
| CHI (Ovarian) | 20 | 100 (72 ± 2%) |
| HX155 (Cervix) | 30 | NS |
| SF1 (Fibroblasts) | 50 | NS |

The lowest concentration of substance P (SP 1-100 μM) which caused significant inhibtion ($P < 0.01$) of $^3$H-thymidine incorporation is also shown (mean ± s.e.m. of control). All assays performed in the presence of 5% FCS. NS = not significant. ND = not done.

and 60 μM respectively (Figure 2). Normal human skin fibroblasts (SF1) showed stimulation (132–155% control $P<0.01$) of DNA synthesis at 1–20 μM D-Phe$^5$SP, however ≥40 μM caused inhibition (65 ± 4% control $P<0.01$). The $IC_{50}$ was 50 μM (Table I).

Substance P (SP) itself had no significant growth inhibitory effect in 5/8 cell lines, while 3/8 showed inhibition (70–90% control $P<0.01$) at 100 μM. SP 1–100 μM had no significant effect on DNA synthesis in normal human skin fibroblasts (SF1) (Table I).

SP (100 μM), bombesin (1 μM), vasopressin (1 μM) and IGF-1 (70 nM) were tested in combination with D-Phe$^5$SP (1–100 μM), to see if they were able to reverse the inhibitory effect of the analogue. No reversal was seen in 3/3 SCLC CLs or 1/1 squamous lung carcinoma CL. This was further investigated in SCLC CL HX149 where inhibition ($P<0.01$) at 20 μM D-Phe$^5$SP in RPMI-1640 supplemented with 5% FCS (56 ± 5% control) and 5 μM in unsupplemented RPMI-1640 (68 ± 2% control) was not reversed by bombesin (1–1,000 nM), vasopressin (1–1,000 nM) or SP (1–100 μM) (data not shown).

### *Inhibition of growth as assessed by cell number*

One week's exposure to 20, 50 or 100 μM D-Phe$^5$SP resulted in the growth inhibition of SCLC CLs HC12 and ICR-SC155. Increase in cell number was significantly inhibited ($P<0.01$) by 20 μM (78 ± 1% and 32 ± 0.4% control respectively), while 50 and 100 μM caused cell number to fall below the initial cell inoculum (Figure 3). In contrast 50 μM D-Phe$^5$SP had no effect on the cell number of confluent cultures of normal human skin fibroblasts, however 100 μM caused inhibition to 83 ± 2% control ($P<0.01$) (Figure 3).

### *Effect of short term exposure to 100 μM analogue*

HC12 viable cell number dropped on exposure to 100 μM D-Phe$^5$SP for 1–4 h. After 1 h the treated cell number fell to 55 ± 4% of the initial inoculum, and by 4 h it was 1 ± 0.1% (Figure 4a). The control cell number recoverable over 4 h was 78 ± 4–90 ± 9% of the initial inoculum.

Surviving treated and control cells were washed and cultured in fresh medium without D-Phe$^5$SP. After 1 week the control cells had grown to 1706 ± 51–2530 ± 59% of the initial cell number. Cells pre-exposed to D-Phe$^5$SP for 1 h showed similar growth to controls. However, cells which had survived 2, 3 and 4 h exposure had a significantly ($P<0.01$) reduced ability to grow, with cell number increases of 647 ± 20%, 224 ± 4% and 335 ± 7% respectively (Figure 4b). The same effects were found in two separate experiments, both carried out on the HC12 cell line.

**Figure 2** D-Phe$^5$SP (1–100 μM) effect on DNA synthesis of HC12 (SCLC CL) in the presence of 5% FCS (■), 10% FCS (▲), and 20% FCS (▼). Results expressed as % control incorporation (mean ± s.e.m.).

**Figure 3** Inhibition of growth of SCLC CLs HC12 and ICR-SC155 after 1 week in the presence of 20, 50 or 100 μM D-Phe$^5$SP. Effect of 3 days exposure to 50 and 100 μM D-Phe$^5$SP on cell number of confluent cultures of normal human skin fibroblasts (SF1). Results expressed a % control cell number (mean ± s.e.m.). *Significant inhibition $P<0.01$.

**Figure 4** **a**, HC12 viable cell number recovered after 1–4 h exposure to PBS (▲) or 100 μM D-Phe$^5$SP (■), expressed as % of initial inoculum of $10^6$ cells (mean ± s.e.m. of triplicate counts). Surviving cells from each time point were seeded at 8.5 × $10^3$ $ml^{-1}$ and grown for 1 week in the absence of analogue. **b**, Histogram showing growth of control (C) and treated (T) cells after 1, 2, 3 or 4 h pre-exposure to 100 μM D-Phe$^5$SP. Cell counts expressed as % growth increase from original cell number (mean ± s.e.m. of triplicate counts). *Significant inhibition ($P<0.01$) compared to control.

### *D-Phe$^5$SP effect on colony forming efficiency (CFE) of cell lines and normal human bone marrow*

D-Phe$^5$SP caused dose dependent inhibition of colony formation ($P<0.01$) in 3/3 SCLC CLs and 1/1 squamous lung carcinoma CL, while substance P had no effect (Table II). Significant inhibition was seen at D-Phe$^5$SP concentration as low as 1 $\mu$M.

Three normal human bone marrow samples showed variable inhibition ($P<0.01$) of colony formation in response to D-Phe$^5$SP. Two of three had an $IC_{50}$ of 30 $\mu$M, while the third had an $IC_{50}$ of 80 $\mu$M. However the degree of inhibition was less than that seen in HC12 under the same growth conditions (Table II). In 20% FCS with GMCSF, 1 $\mu$M D-Phe$^5$SP had no effect on the CFE of HC12, whereas this concentration had caused inhibition (31 ± 2%, $P<0.01$) in medium with 5% FCS.

**Table II** % colony formation (mean ± s.e.m. of control) in the presence of 0–100 $\mu$M D-Phe$^5$SP and 0–100 $\mu$M substance P (SP)

| *Cell line* | *D-Phe$^5$SP $\mu$M* 0 | 1 | 10 | 50 | 100 | *SP $\mu$M* 0–100 |
|---|---|---|---|---|---|---|
| HC12 | 100±14 | 31±2[a] | 5±2[a] | 2±1[a] | 0[a] | NS |
| ICR-SC112 | 100±11 | 8±2[a] | 0[a] | 0[a] | 0[a] | NS |
| HX149 | 100±8 | 93±14 | 0[a] | 0[a] | 0[a] | NS |
| NCI-H226 | 100±13 | 102±16 | 16±2[a] | 20±4[a] | 7±2[a] | NS |
| BM 1 ■ | 100±5 | 120±12[b] | 88±5 | 83±2 | 30±1[a] | ND |
| BM 2 ■ | 100±5 | 98±9 | 70±8[a] | 31±3[a] | 20±3[a] | ND |
| BM 3 ■ | 100±5 | 96±5 | 87±4 | 11±3[a] | 7±2[a] | ND |
| HC12 ■ | 100±8 | 123±6 | 19±1[a] | 2±0.6[a] | 0.6±0.6[a] | ND |

Assays performed in RPMI 1640 medium with 5% FCS except bone marrow (BM) and HC12 assays (■) which were in RPMI 1640 supplemented with 20% FCS and 100 ng GM-CSF $ml^{-1}$. [a]Significant inhibition $P<0.01$; [b]Significant stimulation $P<0.05$; NS = not significant; ND = not done.

### Discussion

This study confirms previous reports of the potent antiproliferative effects of [D-Arg$^1$, D-Phe$^5$, D-Trp$^{7,9}$, Leu$^{11}$]substance P (D-Phe$^5$SP) on small cell lung cancer (SCLC) cell lines (Woll & Rozengurt, 1988*b*; Woll & Rozengurt, 1990) (Figure 1). In addition, we have demonstrated equivalent inhibition of DNA synthesis in fresh SCLC tumour cells from a previously untreated patient, a squamous lung carcinoma cell line, a squamous cervical carcinoma cell line, and two ovarian carcinoma cell lines (Table I).

Previous studies have focused on the ability of the SP analogues to antagonise interaction of neuropeptide growth factors, particularly bombesin, with cell surface receptors (Woll & Rozengurt, 1988*a*; Woll & Rozengurt, 1988*b*; Takuwa *et al*., 1990). Squamous lung carcinoma cell line NCI-H226 lacks detectable bombesin receptors (Moody *et al*., 1983) and appears to be slightly less sensitive to the analogue than the SCLC cell lines. However growth was undoubtedly inhibited (Table II). Exogenous bombesin was not able to reverse D-Phe$^5$SP inhibition of DNA synthesis in the three SCLC cell lines examined, which confirms previous suggestions that the analogue is not working solely by competition for binding to the bombesin receptor (Takuwa *et al*., 1990). This is in contrast with results in Swiss 3T3 cells, where the inhibitory effects of SP analogues can be reversed by excess gastrin releasing peptide (GRP) (Woll & Rozengurt, 1988*b*), and suggests that the mechanism of inhibition is different in the SCLC cells. Similarly, we saw no reversal of inhibition in the presence of vasopressin or IGF1. Increasing serum concentration does partially reverse the inhibitory effect of the analogue, however even in the presence of 20% FCS DNA synthesis and cell growth are almost completely blocked (Figure 2 and Table II).

SP analogue [D-Arg$^1$, D-Pro$^2$, D-Trp$^{7,9}$, Leu$^{11}$]substance P (DAPTL-SP) is reported to have a cytostatic effect in SCLC CLs, with reversal of growth inhibition after washing and reculturing in medium without the analogue (Woll & Rozengurt, 1988*b*). We observed a significant reduction in viable cell number following brief exposure to D-Phe$^5$SP 100 $\mu$M, and the growth potential of the surviving cells was not completely restored on removal of the analogue (Figure 4b). This difference in reversibility may simply reflect the reported increased potency of D-Phe$^5$SP over DAPTL-SP (Woll & Rozengurt, 1988*b*), or they may be inhibiting growth through different mechanisms.

In general D-Phe$^5$SP was less potent when tested against normal human skin fibroblasts (Figure 3) and human bone marrow cells (Table II) than tumour cells. Comparing the growth inhibitory effects of D-Phe$^5$SP against HC12 and human bone marrow samples which were grown under identical assay conditions, colony formation was consistently higher by the bone marrows than the tumour cells, even at 100 $\mu$M. This differential effect between normal and tumour cells could be due to the number or type of receptors expressed on the cell membrane.

In summary D-Phe$^5$SP shows antitumour effects against several different tumour cell types, including SCLC, squamous lung carcinoma, ovarian and squamous cervical carcinoma, with less growth inhibition seen against normal human skin fibroblasts and bone marrow. There is substantial cell death after 2–4 h exposure to 100 $\mu$M D-Phe$^5$SP, and surviving cells exhibit growth inhibition. These results suggest that D-Phe$^5$SP merits further study as a potential novel antitumour agent. We are currently investigating the mechanism of action and *in vivo* activity.

### References

BEPLER, G., CARNEY, D.N., GAZDAR, A.F. & MINNA, J.D. (1987). *In vitro* growth inhibition of human small cell lung cancer by physalaemin. *Cancer Res*., **47**, 2371.

BEPLER, G., ZEYMER, U., MAHMOUD, S. & 7 others (1989). Substance P analogues function as bombesin receptor antagonists and inhibit small cell lung cancer clonal growth. *Peptides*, **9**, 1367.

CARNEY, D.N., GAZDAR, A.F., NAU, M. & MINNA, J.D. (1985). Biological heterogeneity of small cell lung cancer. *Semin Oncol*., **12**, 289.

CORPS, A.N., REES, L.H. & BROWN, K.D. (1985). A peptide that inhibits the mitogenic stimulation of Swiss 3T3 cells by bombesin or vasopressin. *Biochem. J*., **231**, 781.

CUTTITTA, F., CARNEY, D.N., MULSHINE, J. & 4 others (1985). Bombesin-like peptides can function as autocrine growth factors in human small-cell lung cancer. *Nature*, **316**, 823.

EULER, U.S. & GADDUM, J.H. (1931). An unidentified depressor substance in certain tissue extracts. *J. Physiol. (Lond)*., **72**, 74.

EVERARD, M.J., MACAULAY, V.M., MIN, T., MILLAR, J.L. & SMITH, I.E. (1990). Small cell lung cancer cell line from a histologically and immunocytochemically negative bone marrow. *Eur. J. Cancer*, **26**, 767.

HILLS, C.A., KELLAND, L.R., ABEL, G., SIRACKY, J., WILSON, A.P. & HARRAP, K.R. (1989). Biological properties of ten human ovarian carcinoma cell lines: calibration *in vitro* against four platinum complexes. *Br. J. Cancer*, **59**, 527.

JENSEN, R.T., JONES, S.W., FOLKERS, K. & GARDNER, J.D. (1984). A synthetic peptide that is a bombesin receptor antagonist. *Nature*, **309**, 61.

KELLAND, L.R., BURGESS, L. & STEEL, G.G. (1987). Characterization of four new cell lines derived from human squamous carcinoma of the uterine cervix. *Cancer Res*., **47**, 4947.

LAZARUS, L.H., DIAUGUSTINE, R.P., JAHNKE, G.D. & HERNANDEZ, O. (1983). Physalaemin: an amphibian tachykinin in human lung small-cell carcinoma. *Science*, **217**, 79.

LEMBECK, F., FOLKERS, K. & DONNERER, J. (1981). Analgesic effect of antagonists of Substance P. *Biochem. Biophys. Res. Comm*., **103**, 1318.

MACAULAY, V.M., TEALE, J.D., EVERARD, M.J., JOSHI, G.P., SMITH, I.E. & MILLAR, J.L. (1988). Somatomedin-C/insulin-like growth factor-1 is a mitogen for human small cell lung cancer. *Br. J. Cancer*, **57**, 91.

MATSUDA, H., KAWAKITA, K., KISO, Y., NAKANO, T. & KITAMURA, Y. (1989). Substance P induces granulocyte infiltration through degranulation of mast cells. *J. Immunol.*, **142**, 927.

MOODY, T.W., BERTNESS, V. & CARNEY, D.N. (1983). Bombesin-like peptides and receptors in human tumor cell lines. *Peptides*, **4**, 683.

PIERCEY, M.F., SCHROEDER, L.A., FOLKERS, K., XU, J.C. & HORIG, J. (1981). Sensory and motor functions of spinal cord substance P. *Science*, **214**, 1361.

POST, C., BUTTERWORTH, J.F., STRICHARTZ, G.R., KARLSSON, J.A. & PERSSON, C.G.A. (1985). Tachykinin antagonists have potent local anaesthetic actions. *Euro. J. Pharmacol.*, **117**, 347.

ROZENGURT, E. & HEPPEL, L.A. (1975). Serum rapidly stimulates ouabain- sensitive $^{86}Rb^{+}$ influx in quiescent 3T3 cells. *Proc. Natl Acad. Sci. USA*, **72**, 4492.

TAKUWA, N., TAKUWA, Y., OHUE, Y. & 5 others (1990). Stimulation of calcium mobilization but not proliferation by bombesin and tachykinin neuropeptides in human small cell lung cancer cells. *Cancer Res.*, **50**, 240.

WOLL, P.J. & ROZENGURT, E. (1988*a*). Two classes of antagonist interact with receptors for the mitogenic neuropeptides bombesin, bradykinin and vasopressin. *Growth Factors*, **1**, 75.

WOLL, P.J. & ROZENGURT, E. (1988*b*). [D-Arg$^{1}$, D-Phe$^{5}$, D-Trp$^{7,9}$, Leu$^{11}$]Substance P, a potent bombesin antagonist in murine Swiss 3T3 cells, inhibits the growth of human small cell lung cancer cells *in vitro*. *Proc. Natl Acad. Sci. USA*, **85**, 1859.

WOLL, P.J. & ROZENGURT, E. (1990). A neuropeptide antagonist that inhibits the growth of small cell lung cancer *in vitro*. *Cancer Res.*, **50**, 3968.

YACHNIS, A.T., CRAWLEY, J.N., JENSEN, R.T., McGRANE, M.M. & MOODY, T.W. (1984). The antagonism of bombesin in the CNS by Substance P analogues. *Life Sci.*, **35**, 1963.